# Novel potential lncRNA biomarker in B cells indicates essential pathogenic pathway activation in patients with SLE

**DOI:** 10.1136/lupus-2023-001065

**Published:** 2024-04-10

**Authors:** Xinyi Zhu, Yashuo Chen, Zhihua Yin, Yutong Zhang, Yiwei Shen, Dai Dai, Xiaojing Lin, Ling-Hua Zou, Nan Shen, Zhizhong Ye, Huihua Ding, Guojun Hou

**Affiliations:** 1 Shanghai Institute of Rheumatology, Renji Hospital, Shanghai Jiao Tong University School of Medicine (SJTUSM), Shanghai, China; 2 Shenzhen Futian Hospital for Rheumatic Diseases, Shenzhen, Guangdong, China

**Keywords:** Lupus Erythematosus, Systemic, Interferon Type I, B-Lymphocytes

## Abstract

**Objectives:**

Systemic lupus erythematosus (SLE) is a highly heterogeneous disease, and B cell abnormalities play a central role in the pathogenesis of SLE. Long non-coding RNAs (lncRNAs) have also been implicated in the pathogenesis of SLE. The expression of lncRNAs is finely regulated and cell-type dependent, so we aimed to identify B cell-expressing lncRNAs as biomarkers for SLE, and to explore their ability to reflect the status of SLE critical pathway and disease activity.

**Methods:**

Weighted gene coexpression network analysis (WGCNA) was used to cluster B cell-expressing genes of patients with SLE into different gene modules and relate them to clinical features. Based on the results of WGCNA, candidate lncRNA levels were further explored in public bulk and single-cell RNA-sequencing data. In another independent cohort, the levels of the candidate were detected by RT-qPCR and the correlation with disease activity was analysed.

**Results:**

WGCNA analysis revealed one gene module significantly correlated with clinical features, which was enriched in type I interferon (IFN) pathway. Among non-coding genes in this module, lncRNA RP11-273G15.2 was differentially expressed in all five subsets of B cells from patients with SLE compared with healthy controls and other autoimmune diseases. RT-qPCR validated that RP11-273G15.2 was highly expressed in SLE B cells and positively correlated with IFN scores (r=0.7329, p<0.0001) and disease activity (r=0.4710, p=0.0005).

**Conclusion:**

RP11-273G15.2 could act as a diagnostic and disease activity monitoring biomarker for SLE, which might have the potential to guide clinical management.

**WHAT IS ALREADY KNOWN ON THIS TOPIC**

- Systemic lupus erythematosus (SLE) is a highly heterogeneous disease with fluctuating disease course and the identification of a reliable biomarker is of imperative need.
- B cell is considered to have a crucial pathogenic role in SLE as it is causative to the excessive generation of autoantibodies, which characterised lupus.

**WHAT THIS STUDY ADDS**

- We found an SLE gene module in B cells that was significantly correlated with SLE clinical traits in and enriched in interferon pathway.
- We identified lncRNA RP11-273G15.2 as a biologically significant biomarker as it coexpressed with genes critical to SLE in B cells and it outperformed IFN scores to reflect disease activity.

**HOW THIS STUDY MIGHT AFFECT RESEARCH, PRACTICE OR POLICY**

- Our study provides a potential biomarker for SLE by combining bioinformatic analysis with clinical validation, and it might act as an entry point for us to identify therapeutic target and explore disease pathogenesis.

## Introduction

Systemic lupus erythematosus (SLE) is a prototypical autoimmune disease. Both genetic predisposition and environmental factors contribute to its pathogenesis. In patients with SLE, the defect of immune tolerance leads to autoreactive innate and adaptive immune responses. Particularly, the B cell compartment is considered to have an essential pathogenic role in SLE as they are responsible for the overproduction of autoantibodies.^1^ The B cell-targeted therapy, such as belimemab, could effectively attenuate the symptom of SLE, which further demonstrates the pivotal role of B cells in lupus.^2^ Thus, the advance of B cell study can facilitate the development of effective diagnosis, monitoring and therapeutic strategies of SLE. Meanwhile, the identification and validation of a reliable biomarker related to B cell pathophysiology in patients with SLE is of urgent need.

Long non-coding RNAs (lncRNAs) are non-protein-coding RNAs that are >200 nucleotides in length,^3^ some of which are dysregulated in autoimmune diseases and could be causes or consequences of these diseases.^4^ LncRNAs can function as key regulators of gene expression at epigenetic, transcriptional, post-transcriptional and post-translational levels.^4^ For instance, lnc-DC is exclusively expressed in conventional DC cells, and it controls DC differentiation through binding to STAT3.^5^ Recent findings have shown that multiple lncRNAs, such as RP11-2B6,2, MALAT-1 and NEAT1 were engaged in the pathogenesis of autoimmune diseases.^6–8^ Moreover, previous studies indicate that some lncRNAs have the potential to work as biomarkers to reflect SLE activity.^9 10^ However, there are few studies focusing on the lncRNA profiles in B cells of patients with SLE, and B cell expressing lncRNA’s value as biomarker deserve to be evaluated.

The discovery and confirmation of a viable biomarker in SLE investigations remain a major challenge.^11^ Strategies like transcriptional profiling, mass spectrometry-based proteomic profiling, peptidomic profiling, etc, have been adopted to discover disease biomarkers. Traditional methods usually identify individual molecules as biomarkers. In contrast, a bioinformatic strategy named weighted gene coexpression network analysis (WGCNA) can relate gene modules rather than individual genes to external traits, which can reduce the multiple testing problem and is more biologically significant and reliable.^12^ WGCNA constructed gene coexpression networks based on their expression pattern, and it links gene modules to clinical traits, which can help us identify gene modules associated with phenotypes and get insights into the underlying pathogenic processes. Currently, few studies adopted this method to screen lncRNA as a potential biomarker in SLE.

In this study, we integrated WGCNA method, bulk and single-cell RNA-sequencing data to discover and validate a B cell highly expressing lncRNA as potential biomarker, which is pathologically associated with SLE and has the potential to advance the diagnosis, monitoring and therapeutic interventions of SLE.

## Materials and methods

### Study design and study subjects

**Figure 1:**
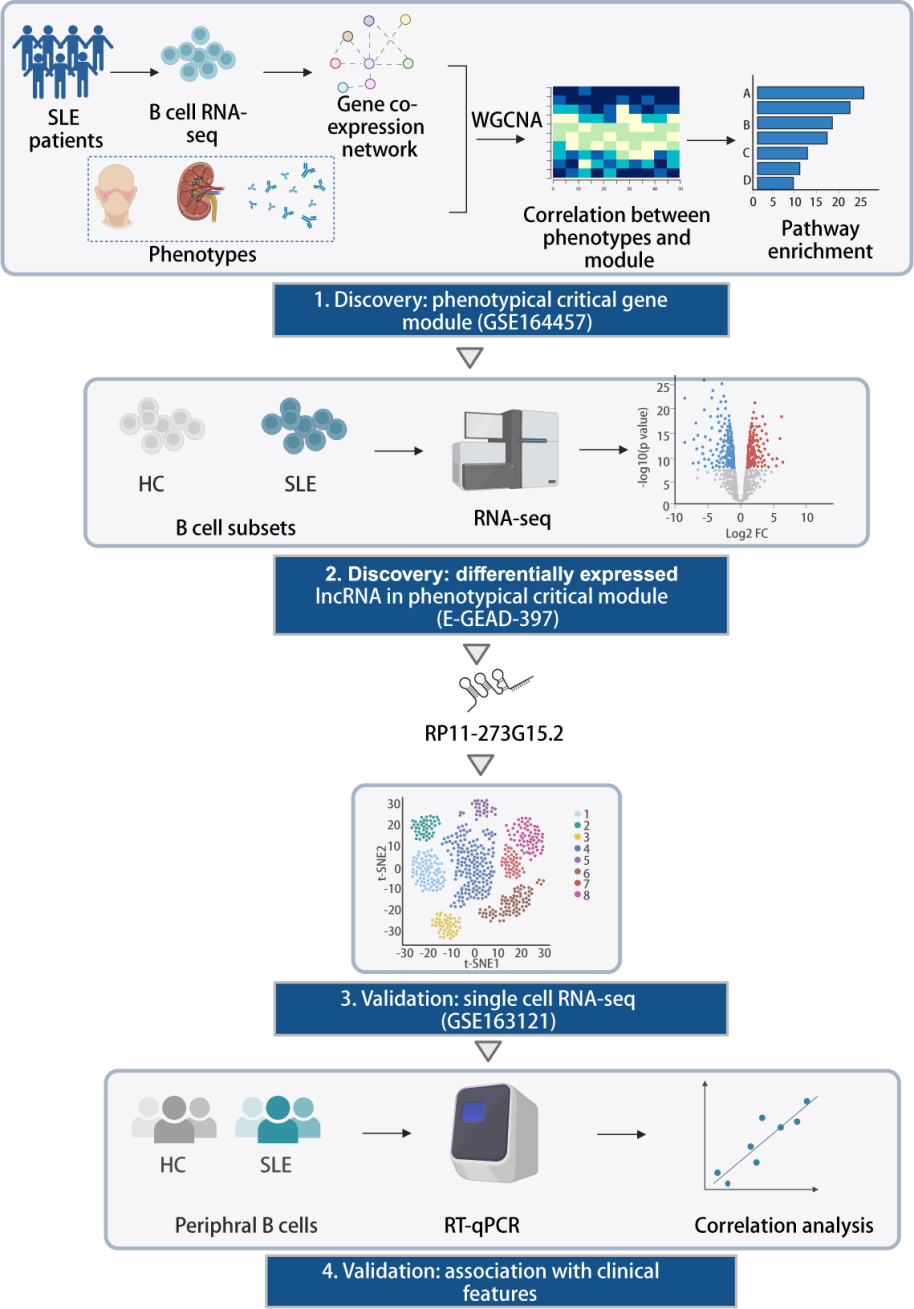
Overall schematics for screening of candidate lncRNAs. (1) WGCNA was applied to high-throughput SLE B-cell transcriptome data (GSE164457) to construction networks with co-expressed genes in the same module. Gene modules were linked to disease phenotypes and the most clinically relevant module was selected to do an enrichment analysis. (2) B cell RNA-seq data (E-GEAD-397) were analysed. LncRNA RP11-273G15.2 was in the critical module and differentially expressed between HCs and SLE. (3) Distribution of RP11-273G15.2 was investigated at a single-cell level (GSE163121). (4) Real-time PCR was used to validate the potential of RP11-273G15.2 as a biomarker in an independent cohort. HCs, healthy control; SLE, systemic lupus erythematosus; WGCNA, weighted gene coexpression network analysis.

In this study, we first employed public B cell RNA-seq data (GSE164457) to perform a WGCNA analysis, from which we discovered a gene module named Saddlebrown as the most prominent module associated with clinical traits, and genes in this module were highly enriched in interferon (IFN) pathway (figure 1). Afterward, we confirmed a candidate lncRNA in this module, RP11-273G15.2, was differentially expressed in 5 B cell subpopulations of patients with SLE compared with healthy controls (HCs) (E-GEAD-397). We selected it as our candidate, and subsequently evaluated its distribution and association with IFN pathway at a single-cell level (GSE163121). Finally, we validated RP11-273G15.2’s potential as a biomarker for SLE in an independent cohort collected in our medical centre (figure 1).

In the validation phase, 51 patients with SLE and 31 age-matched and gender-matched healthy controls were recruited between 2017 and 2018 by the Department of Rheumatology at RenJi Hospital, Shanghai Jiao Tong University School of Medicine. All patients fulfilled the ACR 1997 revised criteria or 2012 SLICC criteria for SLE.^13 14^ All participants were Han Chinese people. Medical records’ review, physician assessment and laboratory test data were retrospectively collected. Disease activity was evaluated according to the SLE Disease Activity Index 2000 (SLEDAI-2K).^15^ The IFN scores were calculated as previously described.^16^ Briefly, we used three representative interferon-stimulated genes (ISGs) to calculate IFN scores: IFIT1, MX1 and IFIT3. To standardise the gene expression level, the mean expression level of IFIT3 detected by RT-qPCR in the healthy control group was calculated and subtracted from the expression level of IFIT3 of each sample in the healthy control group and SLE group, and the remainder was divided by the SD value of IFIT3 in the healthy control group. IFIT1 and MX1 standardised expression levels were computed in the same manner. Finally, the three values were added up to yield the IFN score for each sample. The demographic and clinical information of all patients with SLE is listed in online supplemental table 1.

10.1136/lupus-2023-001065.supp1 Supplementary data

### Sample collection and B cell isolation

Whole blood from participants was collected into EDTA-coated vacutainer tubes. Peripheral blood mononuclear cells were isolated from whole blood by Ficoll density-gradient centrifugation. B cells were further isolated using anti-CD19 magnetic beads (Miltenyi Biotec) and stored in Trizol reagent until use. Total RNA was extracted from B cells.

### RT-qPCR

Quantity and quality of RNA were determined by NanoDrop-2000 spectrophotometer (NanoDrop Technologies, Wilmington, Delaware). RNA was reverse transcribed into cDNA using PrimeScript RT reagent (Vazyme R333-01) in a total volume of 10 ul. The following protocol was used: 50 °C for 15 min, 85 °C for 5 s. qPCR was performed using SYBR qPCR Master Mix (Vazyme Q712-02) by QuantStudio 7 Flex Real-Time PCR System. RPL13A was used as the reference gene. All the primers used are as follows: RP11-273G15.2-F: AGTGTATCACAGCCATTGGGA; RP11-273G15.2-R: AGGACGCTTCGGTCTCTGTA; IFIT1-F: CCACAAGACAGAATAGCCA; IFIT1-R: GCTCCAGACTATCCTTGACC; IFIT3-F: TTCACCTGGAACTTATTCAAG; IFIT3-R: TTTTATGTAGGCCAACAAGTT; MX1-F: GGGTAGCCACTGGACTGA; MX1-R: AGGTGGAGCGATTCTGAG; RPL13A-F: CCTGGAGGAGAAGAGGAAAGAGA; RPL13A-R: TTGAGGACCTCTGTGTATTTGTCAA. Gene expression was calculated using the 2^-ΔΔCt^ method.

### Weighted gene coexpression network analysis

R package WGCNA was used to construct gene coexpression networks. CD19+B cell RNA-seq data of 120 SLE patients were obtained from GEO database through the accession number GSE164457.^17^ Genes with detectable expression levels in more than 20 CD19+B cell samples were included in the WGCNA analysis.^12^ First, samples were clustered according to the Euclidean distance. By inspection, eight outliers were identified and removed by setting the height cut of 3e+06. Next, we choose an empirical value of 12 as the soft threshold to cluster genes into different modules and construct the scale-free gene network. Finally, gene modules were related to clinical features of the subjects and the phenotypic essential module was identified.

### Differentially expressed lncRNA analysis

RNA sequencing data were downloaded from NBDC Human Database through the accession number E-GEAD-397. The counts of lncRNAs were analysed by R package DEseq2. After removing lncRNAs with 0 count in more than 30% of samples, lncRNAs with fold change >2 or <0.5 and FDR <0.05 between healthy controls (HCs) and SLE were selected as differentially expressed lncRNAs. RNA-seq data from 10 SLE patients with low expression of IFN stimulated genes (IFNneg), 10 patients with high expression of IFN-stimulated genes (IFNpos), and 12 HCs were also used to identify differentially expressed lncRNAs from GEO database through the accession number GSE149050.^18^

### Enrichment analysis

Pathway enrichment was performed by Cytoscape V.3.8.2^19^ using the GO ImmuneSystemProcess analysis. The bubble plot was drawn by the R package ggplot2.

### Single-cell analysis

For peripheral B cells, single-cell RNA-seq data were downloaded from GEO database with the accession number GSE163121^20^ and analysed by Seurat V.4.0.6.^21^ Cells with less than 50 genes or more than 12.5% mitochondrial genes detected were removed. Cells with myeloid cell, T cell or NK cell origin were removed. B cell clusters were annotated manually by critical B cell subtype markers like IGHD, IGHG1, CD27, etc.

### Statistical analysis

GraphPad Prism V.9 was used for all statistical analyses and figure plotting. The normality of data was established using Shapiro-Wilk tests. For non-normally distributed data, non-parametric Mann-Whitney U-test was used to make a comparison between the two groups. For the comparison of RP11-273G15.2 expressing cells between HCs and patients with SLE, an unpaired t-test was applied. For correlation analysis, Spearman’s method was employed. The diagnostic and disease activity monitoring performance of the biomarker was assessed using receiver operating characteristic (ROC) curve analysis. Statistical significance was defined as a two-tailed value of p<0.05.

## Results

### Genes in the disease-critical module were enriched in type I IFN pathway

**Figure 2:**
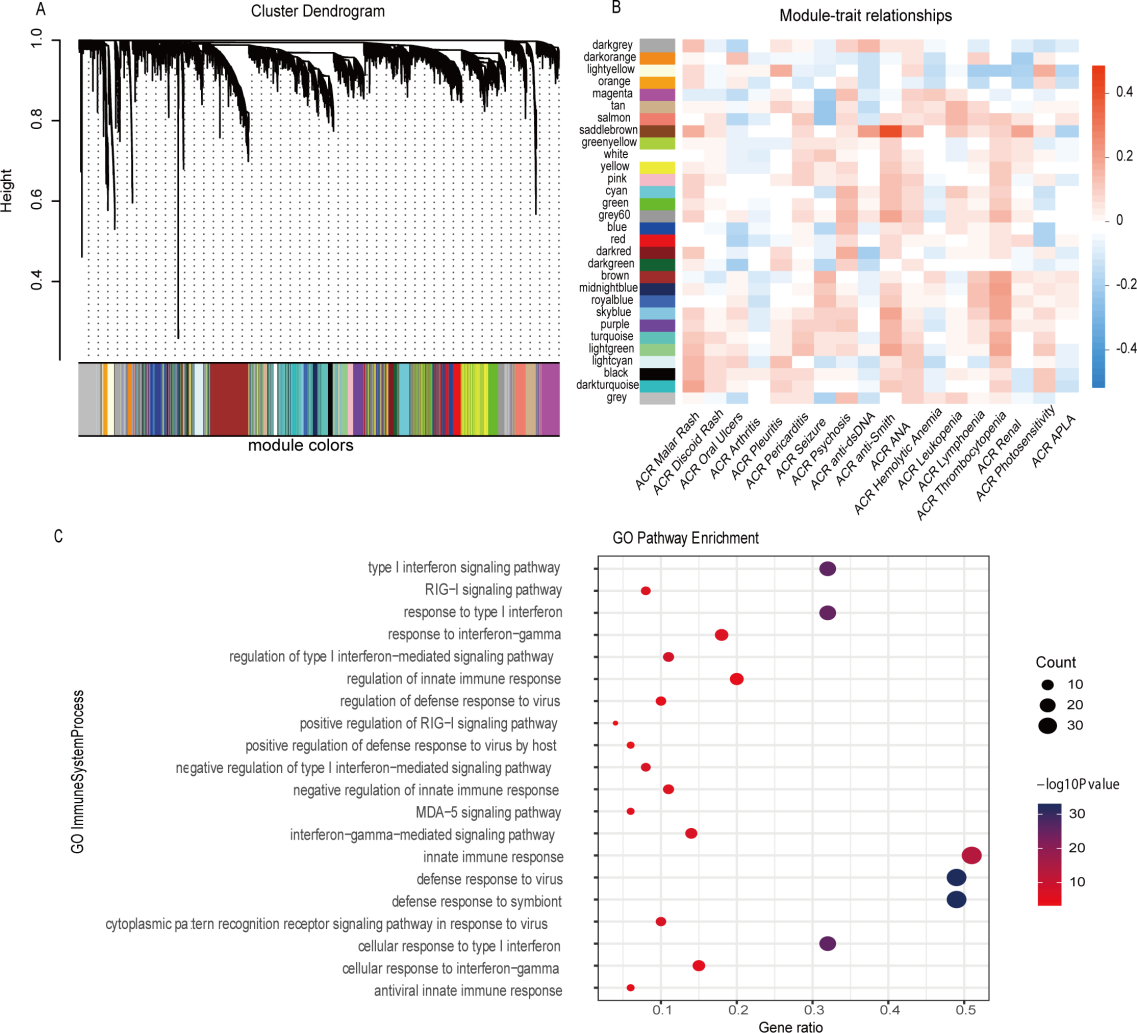
Clustering of genes in B cells of patients with SLE by WGCNA. (A) Dendrogram of clustered gene modules. (B) Heatmap of association between gene modules and phenotypes. Colours reflected correlation coefficient. Blue and red colours represent negatively correlated and positively correlated, respectively. (C) Bubble plot of pathway enrichment of genes in Saddlebrown module. GO pathway terms were displayed in Y axis and X axis displayed gene ratio. Colours reflected p values, whereas bubble size represented gene counts. SLE, systemic lupus erythematosus; WGCNA, weighted gene coexpression network analysis.

To find the B cell critical gene modules that were associated with SLE, we first performed a WGCNA on RNA sequencing data of B cells from 120 patients with SLE (GSE164457).^17^ Genes with detectable expression levels in B cells were applied to WGCNA. In the process of network construction, coexpression similarity between gene pairs was transformed into connection strengths and they formed an adjacency matrix, which was used to define network modules.^22^ In our analysis, all filtered genes in B cells were clustered into 29 modules (figure 2A and online supplemental table 2) with densely connected genes in the same gene module, and the genes that could not be classified in the grey module.

10.1136/lupus-2023-001065.supp2 Supplementary data

To identify which module was critical to SLE, a correlation analysis was performed between these modules and clinical manifestations (figure 2B). Notably, the module named Saddlebrown was the one most significantly correlated with SLE traits, including the presence of malar rash (p=0.037), positivity of anti-dsDNA antibodies (p=0.016), positivity of anti-Smith antibodies (p=1.824E-6) and renal involvement (p=0.022) (online supplemental table 3). There were 71 coexpressed genes in this module, including 4 non-coding RNAs and 67 protein-coding genes. Based on the strong correlation between this module and clinical manifestations, we concentrated on the Saddlebrown module with interest in the following study.

10.1136/lupus-2023-001065.supp3 Supplementary data

The Saddlebrown module was significantly associated with clinical phenotypes, indicating it might relate to essentially biological processes in B cells critical to SLE. To further explore the immunological functions of this module, we performed an enrichment analysis using the genes in this module. As depicted in figure 2C, genes in this module were highly enriched in type I IFN pathway. Based on these observations, we named Saddlebrown module as interferon-associated module. To sum, our rediscovery offered validity of the importance of type I IFN to disease performance. Furthermore, lncRNAs clustered in this module might be related with type I IFN pathway, and their potential as biomarkers deserve to be further validated.

### RP11-273G15.2 was differentially expressed in various B cell subtypes

**Figure 3:**
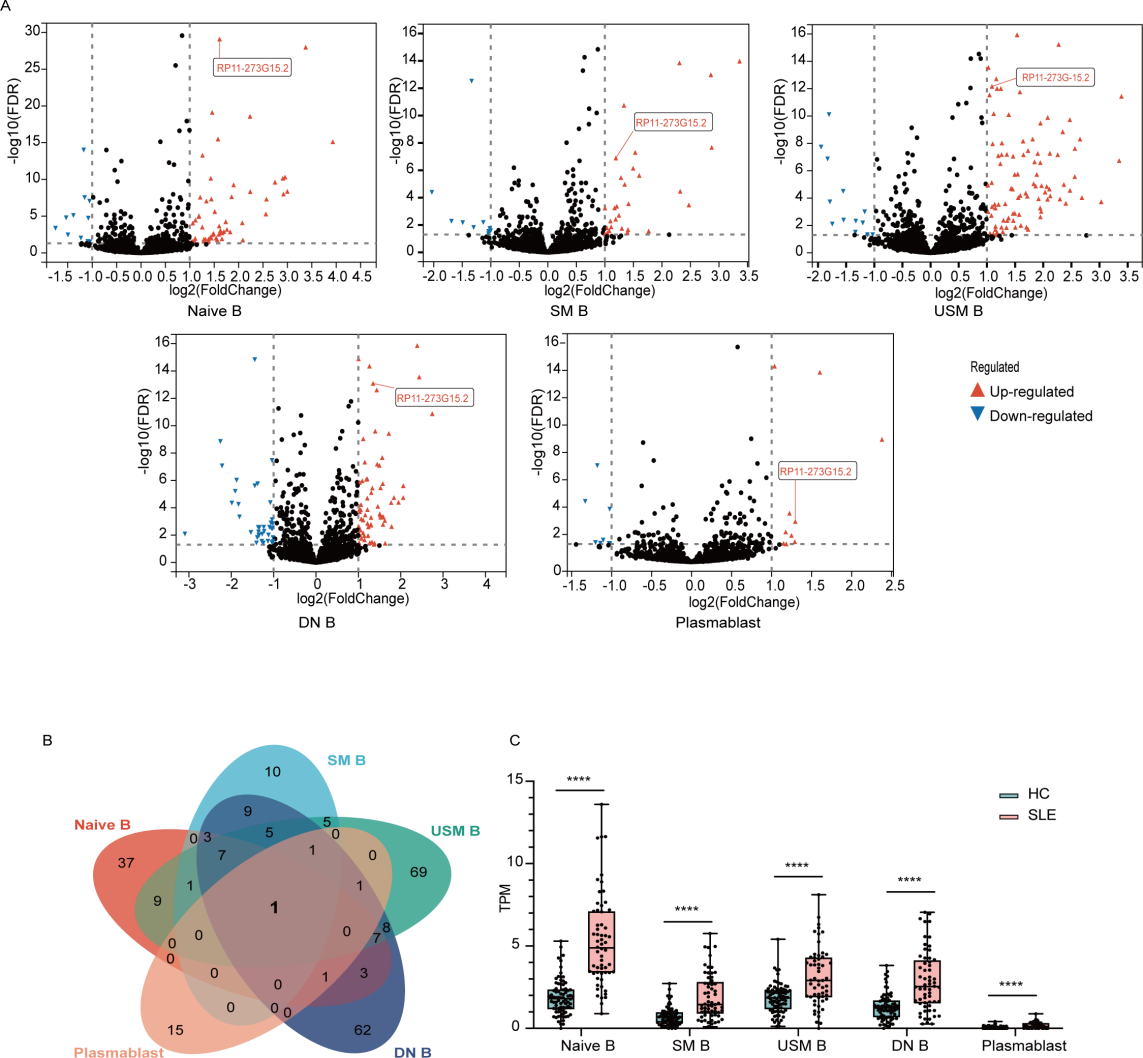
RP11-273G15.2 was differentially expressed in various B cell subsets. (A) Volcano plots indicate lncRNA expression in different B cell subsets. Blue and red colours represent downregulation and upregulation, respectively. (B) Venn diagram indicates the differentially expressed lncRNAs between HCs and SLE. (C) Expression of RP11-273G15.2 in different B cell subpopulations of healthy controls and patients with SLE. Mann-Whitney test was used. ****p<0.0001. HCs, healthy control; SLE, systemic lupus erythematosus.

In order to identify lncRNA biomarkers in SLE patients’ B cells, we further analysed lncRNA transcriptomic landscapes in five different B cell subpopulations from NBDC Human Database (E-GEAD-397).^23^ As shown in figure 3A, there were 108, 69, 19, 42 and 114 differentially expressed lncRNAs in double negative B cells, naïve B cells, plasmablasts, switched memory B cells and unswitched memory B cells, respectively (padj <0.05, fold change >2 or <0.5) (online supplemental table 4). Notably, there was only one differentially expressed lncRNA, RP11-273G15.2, which was also present in the interferon-associated module, overlapped among these 5 B cell subpopulations (figure 3B,C).

10.1136/lupus-2023-001065.supp4 Supplementary data

We also investigated other two autoimmune diseases: rheumatoid arthritis and systemic sclerosis (SSc). As demonstrated in the left panel of online supplemental figure 1A–E, the levels of RP11-273G15.2 were significantly increased in double-negative B cells, naïve B cells, unswitched memory B cells and plasmablasts of patients with SLE compared with those in RA and SSc patients, with RP11-273G15.2 between SLE and RA patients in switched memory B cells as an exception (online supplemental figure 1C). By ROC analysis, RP11-273G15.2 could discriminate patients with SLE from RA and patients with SScin all subsets of B cells except RA patients in switched memory B cells. The greatest area under the curves (AUCs) were in naïve B cell for both of RA (AUC=0.7924, p<0.0001) and SSc (AUC=0.7854, p<0.0001) (right panel of online supplemental figure 1A–E). It suggested that B cell RP11-273G15.2 could be a potential diagnostic biomarker for SLE with its discriminative ability in SLE versus RA, SSc and HC.

As mentioned above, coexpressed genes were clustered into the same module. Coding genes in the interferon associated module were enriched in type I IFN pathway (figure 2C), which indicated RP11-273G15.2 was coexpressed with ISGs in B cells. To further confirm its relationship with type I IFN pathway, we analysed RP11-273G15.2 expression from patients with SLE with low expression of ISGs (IFNneg), SLE patients with high expression of ISGs (IFNpos), and HCs (GSE149050).^18^ Consistent with the results of WGCNA analysis, RP11-273G15.2 was highly expressed in B cells from IFN positive patients while there was no significant difference between HCs and IFN negative patients (online supplemental figure 2A, B), and this result was further confirmed in our independent cohort (online supplemental figure 2C). Consequently, we chose RP11-273G15.2 as our candidate biomarker.

### Analysis of RP11-273G15.2 distribution at a single-cell level

**Figure 4:**
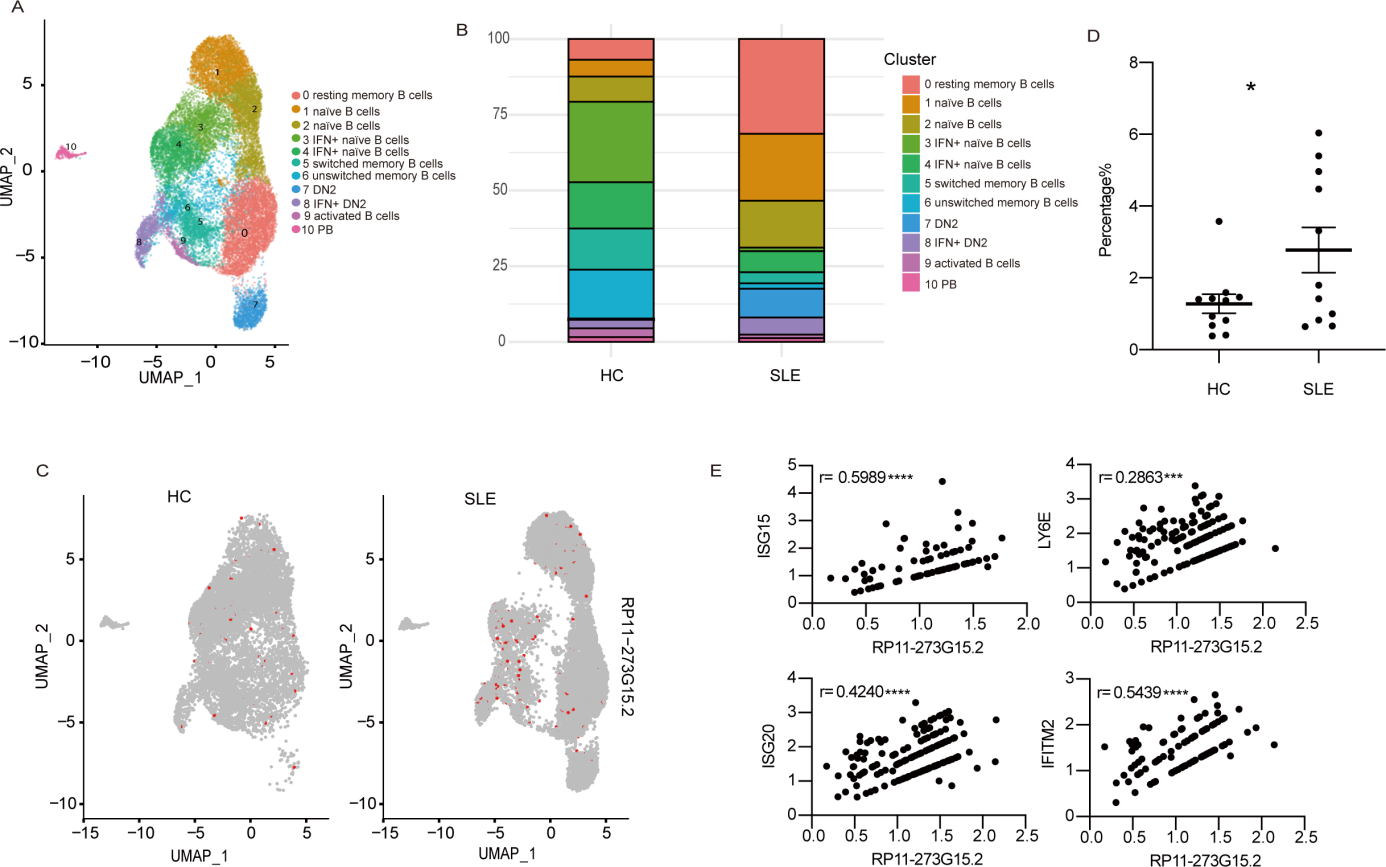
RP11-273G15.2 expression at a single-cell level. (A) UMAP displaying different B cell subsets. (B) Percentages of different subpopulations of B cells were shown. (C) Feature plot of RP11-273G15.2 expression in HC and SLE B cells. (D) The distribution of RP11-273G15.2 expression across different B cell clusters in patients with HC and SLE. Analysis was done by unpaired t-test. (E) Correlation of RP11-273G15.2 levels with different ISGs on a single cell. Correlation analysis was done by Spearman’s method. *p<0.05; **p<0.01; ****p<0.0001. DN2, double-negative 2; HCs, healthy control; IFN, interferon; IFN+DN2, double-negative two with interferon signature; IFN+Naïve, naïve with interferon signature; ISGs, interferon-stimulated genes; PB, plasmablasts; SLE, systemic lupus erythematosus; UMAP, uniform manifold approximation and projection.

As B cells are divided into various subtypes, transcriptome data at single-cell resolution can yield the expression pattern of RP11-273G15.2 in different subpopulations of B cells and provide insights into the pathogenic processes of lupus. Single-cell RNA-sequencing data of SLE patients’ and HCs’ B cells (GSE163121) were analysed by Seurat4.0.6. Peripheral B cells were clustered into 11 groups (figure 4A,B, online supplemental figures 3 and 4). As shown in figure 4C, more SLE peripheral B cells expressed RP11-273G15.2 than HC B cells, and in general, there was a higher percentage of SLE B cells that expressed RP11-273G15.2 than HC B cells (figure 4D). Moreover, in accordance with bulk RNA-seq data, the expression levels of RP11-273G15.2 were positively correlated with ISGs in single cell level of SLE B cells (figure 4E).

### RP11-273G15.2 is associated with disease activity and clinical traits

**Figure 5:**
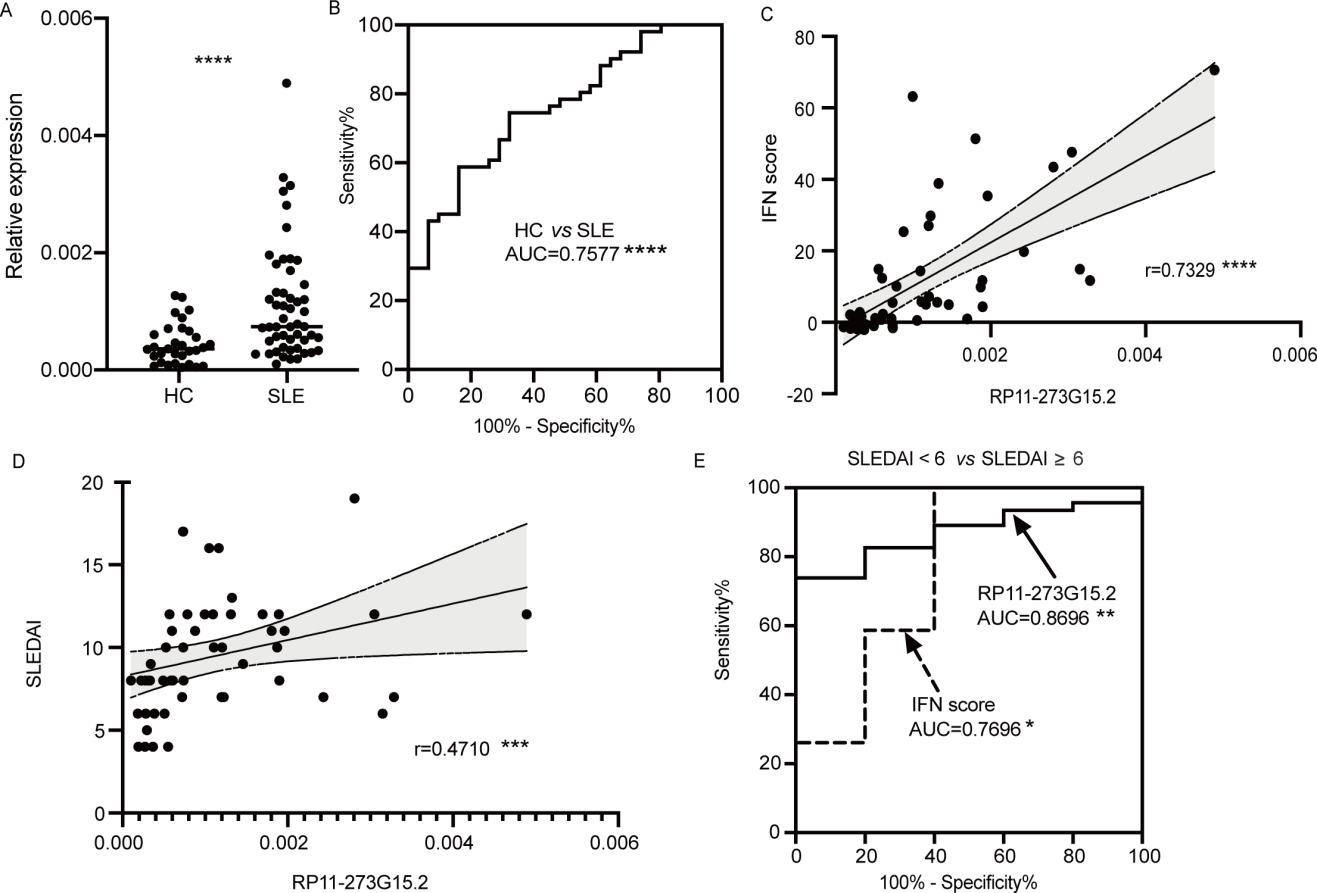
Correlation of RP11-273G15.2 expression in B cells with IFN scores and disease activity. (A) The expression level of RP11-273G15.2 in B cells of healthy controls (HCs) and SLE patients. Mann-Whitney test was used. (B) ROC curve of RP11-273G15.2 level in discriminating SLE from HC. (C–D) The expression level of RP11-273G15.2 in SLE patients’ B cell was positively correlated with (C) IFN scores and (D) SLEDAI scores. (E) ROC curve of RP11-273G15.2 expression level and IFN score in separating SLE patients with SLEDAI-2K scores<6 from those with SLEDAI-2K scores≥6. Spearman’s method was used for correlation analysis. *p<0.05; **p<0.01; ***p<0.001; ****p<0.0001. AUC, area under the curve; IFN, interferon; ROC, receiver operating characteristic; SLE, systemic lupus erythematosus; SLEDAI, SLE Disease Activity Index.

To validate whether RP11-273G15.2 could act as a biomarker, we evaluated its performance in an independent cohort of n=82 (HC=31, SLE=51). In consistent with previous findings, RP11-273G15.2 was highly expressed in SLE patients’ B cells compared with HCs (figure 5A). Its performance as a diagnostic biomarker was assessed by the receiver operating curve (ROC) analysis. As shown in figure 5B, AUC value was 0.7577 (95% CI 0.6544 to 0.8611; p<0.0001) for SLE versus HCs. For the purpose to evaluate its ability to reflect the activation status of IFN signalling pathway. Expression levels of 3 ISGs, IFIT1, MX1 and IFIT3 in B cells were detected by RT-qPCR. The results showed that RP11-273G15.2 expression levels were positively correlated with these 3 ISGs (online supplemental figure 5A–C). Then individual IFN-scores were calculated based on these 3 ISGs. We found that there was a statistically significant correlation between RP11-273G15.2 expression levels and IFN-scores (p<0.0001, figure 5C). To further explore the value of RP11-273G15.2 to indicate disease activity, we performed a correlation analysis between RP11-273G15.2 levels and SLEDAI scores. As demonstrated in figure 5D, RP11-273G15.2 levels were highly correlated with SLEDAI scores, supporting its role as an indicator of disease activity. Remarkably, RP11-273G15.2 outperformed IFN score as a biomarker to reflect disease activity, as AUC value was 0.8696 (95% CI 0.7635 to 0.9757; p =0.0071) for RP11-273G15.2 and 0.7696 (95% CI 0.5002 to 1.000; p = 0.0495) for IFN score (figure 5E). Besides, IFN scores and SLEDAI scores were also positively correlated (online supplemental figure 5D), in accordance with previous findings.^16^

We also compared the expression level of RP11-273G15.2 in SLE patients with or without a specific clinical feature. As summarised in table 1, RP11-273G15.2 levels were associated with multiple clinical features and could be used to indicate the presence of these features, including vasculitis (p=0.004), thrombocytopenia (p=0.010), lymphocytopenia (p=0.007) and leukocytopenia (p=0.006). Moreover, RP11-273G15.2 expression level was correlated with the presence of some autoantibodies, including anti-Sm (p=0.008), anti-U1RNP (p=0.009), anti-ribosomal P protein (p=0.004) antibodies, but no significant correlation was detected for complements, erythrocyte sedimentation rate and autoantibodies including ANA, anti-dsDNA, anti-SSA-Ro52, anti-SSA-Ro60, anti-SS-B (La), anti-histone and anti-nucleosome. To note, both of anti-Sm and anti-ribosomal P protein antibodies are highly specific diagnostic biomarkers of lupus and are currently used in clinical practice.^11^ Taken together, RP11-273G15.2 could reflect multiple aspects of patients with SLE, including IFN signalling status, disease activity, symptoms and autoantibody positivity.

**Table 1:** RP11-273G15.2 expression levels in patients with SLE with or without clinical features

	SLE	Clinical features present	SLE	Clinical features absent	P value
	N	Median (IQR, 10^−3^)	N	Median (IQR, 10^−3^)	
Clinical features at sample collection					
Vasculitis	3/51 (5.88%)	2.81 (2.62, 2.98)	48/51 (94.12%)	0.73 (0.38, 1.27)	0.004**
Malar rash	17/51 (33.33%)	1.16 (0.72, 1.87)	34/51 (66.67%)	0.67 (0.35, 1.21)	0.050
Discoid rash	9/51 (17.65%)	0.59 (0.34, 1.46)	42/51 (82.35%)	0.76 (0.50, 1.32)	0.952
Oral ulcer	1/51 (1.96%)	3.28 (3.28, 3.28)	50/51 (98.04%)	0.74 (0.44, 1.32)	0.078
Thrombocytopenia	8/51 (15.69%)	1.55 (0.95, 2.93)	43/51 (84.31%)	0.72 (0.35, 1.27)	0.010*
Lymphocytopenia	22/51 (43.14%)	1.18 (0.76, 1.93)	29/51 (56.86%)	0.59 (0.37, 0.99)	0.007**
Anaemia	32/51 (62.75%)	0.83 (0.54, 1.79)	19/51 (37.25%)	0.60 (0.36, 1.32)	0.250
Leukocytopenia	11/51 (21.57%)	1.81 (1.18, 2.20)	40/51 (78.43%)	0.73 (0.35, 1.16)	0.006**
Renal involvement	24/51 (47.06%)	0.73 (0.44, 1.14)	27/51 (52.94%)	0.99 (0.45, 1.58)	0.533
Arthritis	8/51 (15.69%)	1.21 (0.59, 1.38)	43/51 (84.31%)	0.73 (0.38, 1.51)	0.452
Serositis	9/51 (17.65%)	0.73 (0.52, 1.90)	42/51 (82.35%)	0.76 (0.44, 1.27)	0.584
Neuropsychiatric	3/51 (5.88%)	0.78 (0.65, 0.92)	48/51 (94.12%)	0.74 (0.38, 1.58)	0.865
Photosensitivity	4/51 (7.84%)	1.15 (0.64, 1.54)	47/51 (92.16%)	0.73 (0.44, 1.39)	0.853
Laboratory test					
C3<0.9 g/L	13/50 (26.00%)	0.74 (0.57, 1.11)	37/50 (74.00%)	0.73 (0.39, 1.46)	0.799
C4<0.1 g/L	31/50 (62.00%)	0.73 (0.43, 1.13)	19/50 (38.00%)	1.20 (0.44, 1.85)	0.219
ANA	51/51 (100.00%)	0.74 (0.44, 1.39)	0/51 (0.00%)	–	–
Anti-dsDNA	38/51 (74.51%)	0.94 (0.58, 1.51)	13/51 (25.49%)	0.52 (0.39, 1.20)	0.300
Anti-Sm	12/50 (24.00%)	1.38 (1.07, 2.20)	38/50 (76.00%)	0.73 (0.35, 1.18)	0.008**
Anti-U1RNP	23/50 (46.00%)	1.20 (0.60, 1.84)	27/50 (54.00%)	0.72 (0.29, 1.08)	0.009**
Anti-SSA-Ro52	28/50 (56.00%)	0.76 (0.44, 1.26)	22/50 (44.00%)	0.91 (0.58, 1.75)	0.584
Anti-SSA-Ro60	27/50 (54.00%)	0.99 (0.50, 1.38)	23/50 (46.00%)	0.72 (0.45, 1.51)	0.514
Anti-SS-B (La)	8/50 (16.00%)	0.76 (0.36, 1.02)	42/50 (84.00%)	0.81 (0.52, 1.58)	0.427
Anti-ribosomal P protein	16/51 (31.37%)	1.56 (0.76, 2.62)	35/51 (68.63%)	0.61 (0.35, 1.10)	0.004**
Anti-histone	13/51 (25.49%)	1.09 (0.33, 1.33)	38/51 (74.51%)	0.74 (0.50, 1.38)	0.931
Anti-nucleosome	33/51 (64.71%)	0.73 (0.39, 1.33)	18/51 (35.29%)	0.94 (0.50, 1.38)	0.783
ESR>20 mm/h(woman), >15 mm/h (man)	43/51 (84.31%)	0.74 (0.44, 1.58)	8/51 (15.69%)	0.67 (0.40, 1.25)	0.533

P values were for comparing RP11-273G15.2 levels in patients with or without clinical features by Mann-Whitney Wilcoxon U test, *p<0.05; **p<0.01;***p<0.001; ****p<0.0001.

ANA, antinuclear antibody; C3, complement 3; C4, complement 4; ESR, erythrocyte sedimentation rate; SLEDAI, Systemic Lupus Erythematosus Disease Activity Index; U1RNP, U1 ribonucleoprotein.

## Discussion

In this study, we integrated high-throughput B-cell transcriptome data of patients with SLE, WGCNA analysis, single-cell RNA-sequencing data of SLE patients’ B cells to discover potential lncRNA biomarkers and performed validation in an independent cohort. We found that lncRNA RP11-273G15.2, dysregulated in SLE patients, was highly correlated with IFN scores and SLEDAI scores, which might help SLE diagnosis, disease activity monitoring and contribute to the development of clinical management of patients with SLE.

Traditional methods that discover SLE biomarkers usually rely on differential gene expression analysis. However, it would generate many candidates, and it is still a challenge to detect a biologically relevant and reliable biomarker. We applied WGCNA to high-throughput B-cell transcriptome data of patients with SLE, and rather than a single gene, a cluster of genes was found to be substantially related to SLE clinical manifestations. These genes were highly enriched in type I IFN pathway. Actually, type I IFN can impact B-cell functions involving in SLE in multiple ways, including effects on receptor engagement, toll-like receptor expression, cell migration, antigen presentation, cytokine responsiveness, cytokine production, survival, differentiation and class-switch recombination.^24–29^ In the following investigation, we focused on lncRNAs in this gene cluster, which were more pathogenically significant and have greater potential to be reliable biomarkers of SLE. Resultantly, the correlation between SLE phenotypes and gene expression patterns in B cells was established, and a phenotypic essential gene module was detected.

The disease course of SLE is unpredictable and fluctuating. Due to the occurrence of flares could lead to organ damage and poor disease prognosis in lupus patients, the monitoring of disease activity and timely interventions are of great importance. In reality, the progression of the disease is determined by disease-critical cell subpopulations, and the dysregulation of key genes in these subpopulations could reflect disease activity. Our results demonstrated that lncRNA RP11-273G15.2 was coexpressed with coding genes critical to SLE and highly expressed in B cell of patients with SLE compared with healthy controls. Furthermore, the data of another cohort illustrated RP11-273G15.2 expression levels were positively correlated with SLEDAI-2K scores, which were taken as the standard measurement of disease activity in clinical practice. This process would accelerate the progress of biomarker discovery and lupus study. However, establishment of the predictive capacity of RP11-273G15.2 as a biomarker still needs to be validated in the larger SLE cohort.

Our findings illuminate that RP11-273G15.2 coexpresses with ISGs; however, the function and mechanism of RP11-273G15.2-mediating SLE pathogenesis are unclear. The bulk of evidence has demonstrated that lncRNA could disrupt the immune homeostasis involving autoimmune disease pathogenesis by affecting disease critical gene expression. Investigating the function and mechanism of RP11-273G15.2 dysfunction would definitely help to dissect the mechanism driving SLE pathogenesis. Using bioinformatic analysis, we preliminary explored the function of RP11-273G15.2. The online platform ENCORI^30^ predicts that it could interact with miR-19a-3p and miR-19b-3p. Overexpression of miR-19a and miR-19b was shown to downregulate the expression of IFN-related genes.^31^ Consequently, RP11-273G15.2 might engage in IFN pathway by acting as a competitive endogenous RNA (ceRNA) to bind miR-19 and prevent its regulatory function. However, these are very preliminary exploration. Future studies are not only tasked with validating whether this lncRNA could act as a biomarker but also with establishing a comprehensive understanding of how this lncRNA drives the SLE process.

Taken together, our study suggested that expression level of RP11-273G15.2 was highly correlated with IFN scores and disease activity in patients with SLE and it might work as a biomarker for SLE diagnosis and disease activity monitoring.

## Data Availability

Data are available in a public, open access repository. All data relevant to the study are included in the article or uploaded as supplementary information.
