# Supplementary material for: Novel potential lncRNA biomarker in B cells indicates essential pathogenic pathway activation in patients with SLE
Source: Lupus Sci Med. 2024 Apr 10;11(1):e001065. doi: 10.1136/lupus-2023-001065 (PMC11015226; doi:10.1136/lupus-2023-001065)
Supplement: Supplementary data [file lupus-2023-001065supp001.pdf]

Supplementary Material

Supplementary Table 1 Demographic and clinical data of all subjects with SLE

**Basic information**	**SLE (n=51)**
Age (years), median (IQR)	31 (20.5 to 37.5)
Female/Male	45/6 (88.24%)
Disease duration (months), median (IQR)	12.17 (2.55 to 49.57)
IFN score, median (IQR)	4.42 (0.59 to 14.63)
SLEDAI-2K, median (IQR)	9 (7 to 12)
**Clinical features at sample collection**	
Vasculitis (+)	3/51 (5.88%)
Malar rash (+)	17/51 (33.33%)
Discoid rash (+)	9/51 (17.65%)
Oral ulcer (+)	1/51 (1.96%)
Thrombocytopenia (+)	8/51 (15.69%)
Lymphocytopenia (+)	22/51 (43.14%)
Anemia (+)	32/51 (62.75%)
Leukocytopenia (+)	11/51 (21.57%)
Renal involvement (+)	24/51 (47.06%)
Arthritis (+)	8/51 (15.69%)
Serositis (+)	9/51 (17.65%)
Neuropsychiatric (+)	3/51 (5.88%)
Photosensitivity (+)	4/51 (7.84%)
**Laboratory test**	
C3 (g/l), median (IQR)	0.53 (0.38 to 0.68)
C4 (g/l), median (IQR)	0.09 (0.06 to 0.11)

ANA (+)	51/51 (100.00%)
Anti-dsDNA (+)	38/51 (74.51%)
Anti-Sm (+)	12/50 (24.00%)
Anti-U1RNP (+)	23/50 (46.00%)
Anti-SSA-Ro52 (+)	28/50 (56.00%)
Anti-SSA-Ro60 (+)	27/50 (54.00%)
Anti-SS-B (La) (+)	8/50 (16.00%)
Anti-ribosomal P protein (+)	16/51 (31.37%)
Anti-histone (+)	13/51 (25.49%)
Anti-nucleosome (+)	33/51 (64.71%)
ESR (mm/h), median (IQR)	49 (29 to 74)

IQR interquartile range, SLEDAI Systemic Lupus Erythematosus Disease Activity Index, C3 complement 3, C4 complement 4, ANA antinuclear antibody, U1RNP U1 ribonucleoprotein, ESR erythrocyte sedimentation rate.

Supplementary Figures

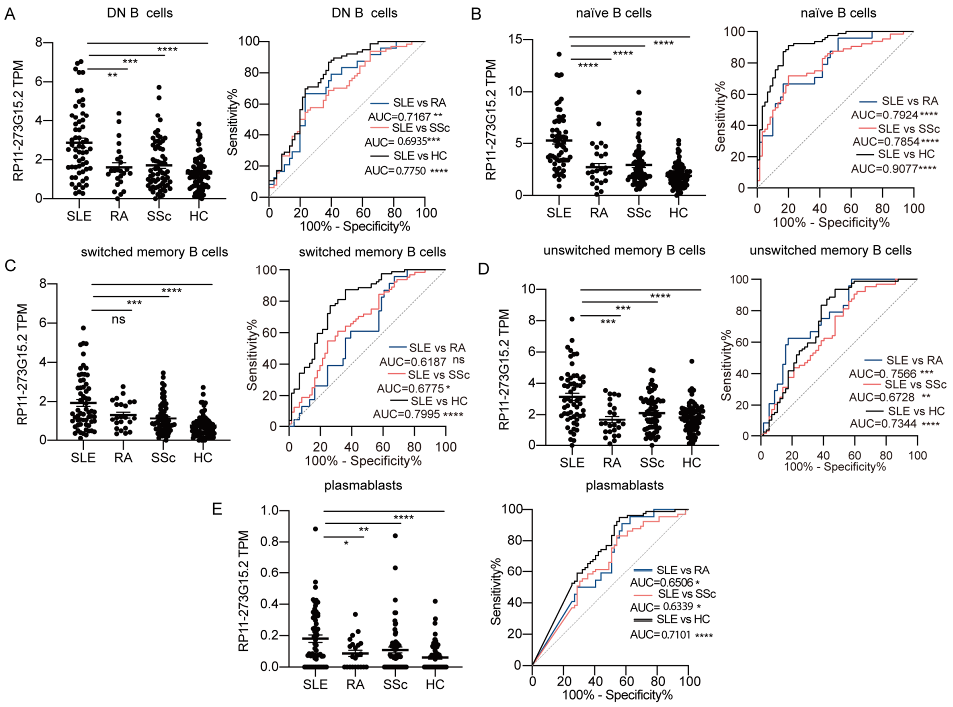

Supplementary Figure 1. RP11-273G15.2 expression levels in different subsets of B cells from patients with distinct disease. RP11-273G15.2 expression levels in DN B cells (A), naïve B cells (B), switched memory B cells (C), unswitched memory B cells (D), and plasmablasts (E) from patients with SLE, RA, SSc or HC (left panel). Mann-Whitney test. ROC curve for RP11-273G15.2 to differentiate SLE from RA, SSc or HC in DN B cells (A), naïve B cells (B), switched memory B cells (C), unswitched memory B cells (D), and plasmablasts (E) (right panel). RA, rheumatoid arthritis; SSc, systemic sclerosis; HC, healthy control; ROC, receiver operating characteristic; AUC, area under the curve. *p<0.05; **p<0.01; ***p<0.001; ****p<0.0001.

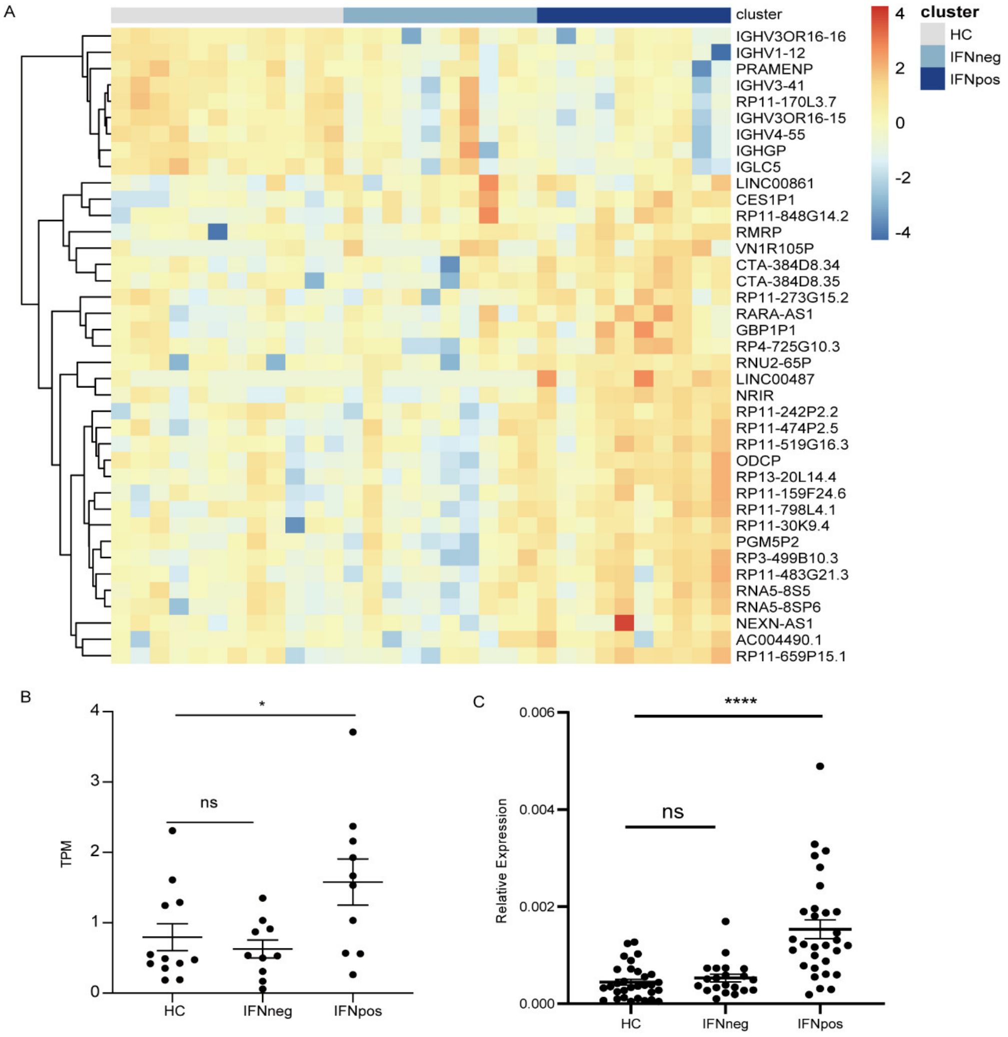

Supplementary Figure 2. RP11-273G15.2 was differentially expressed in IFN-positive B cells. A. Heatmap of differentially expressed lncRNAs between IFN positive SLE patients and healthy controls. Blue and red colors represent downregulation and upregulation respectively. B. TPM of RP11-273G15.2 among groups of HCs, IFN negative patients and IFN positive patients. C. RT-qPCR detects the expression of RP11-273G15.2 among groups of HCs, IFN negative patients and IFN positive patients in our cohort (HC = 31, IFNneg = 21, IFNpos = 30). TPM, transcript per million. Mann-Whitney test was used. Ns: not significant; *p<0.05, ****p<0.0001.

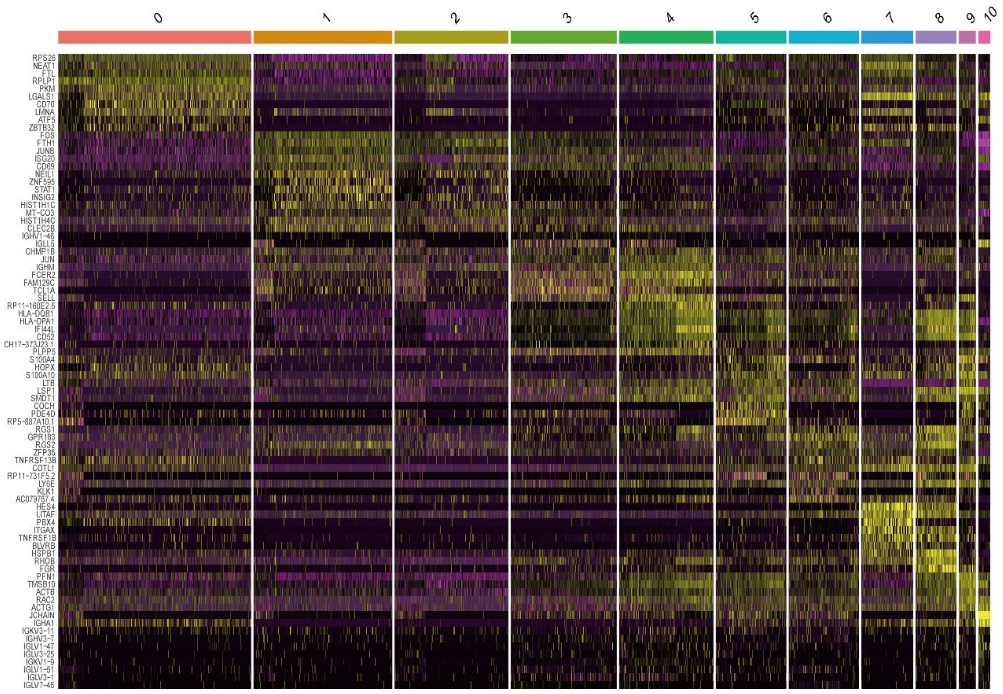

Supplementary Figure 3. Heatmap of top10 marker genes of each cluster.

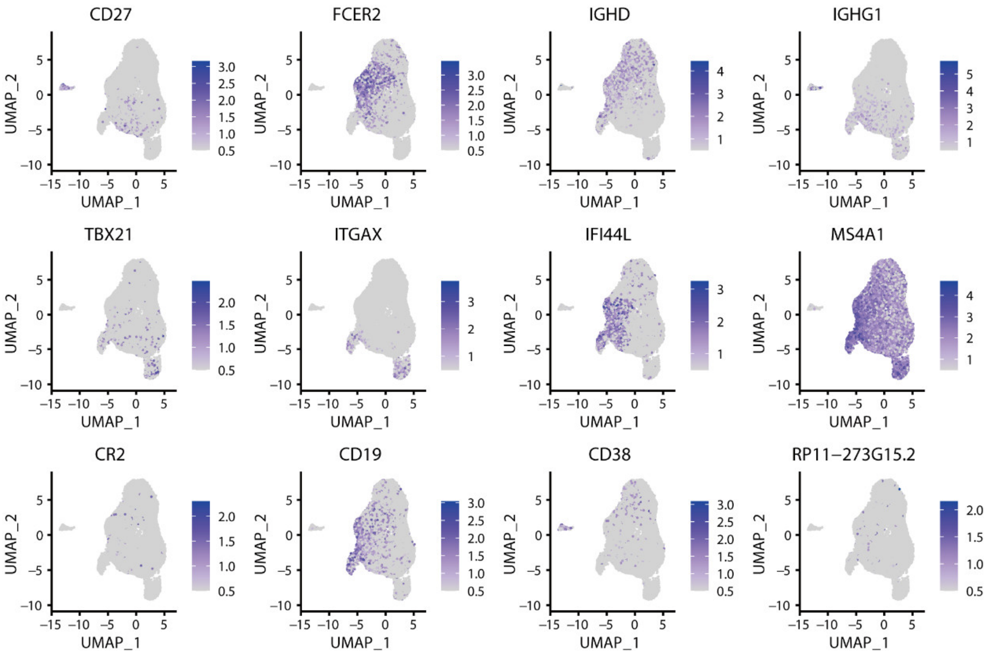

Supplementary Figure 4. Expression patterns of B cell feature genes across distinct cluster.

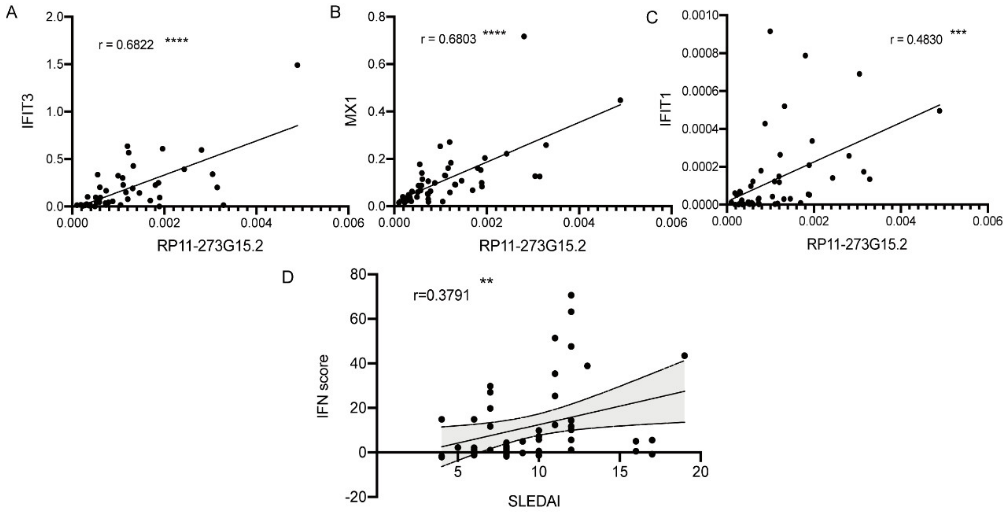

Supplementary Figure 5. Expression levels of RP11-273G15.2 were correlated with ISGs. A. Correlation of RP11-273G15.2 levels with IFIT3, MX1, and IFIT1 levels in peripheral B cells, respectively. B. Correlation of IFN scores in SLE patients with SLEDAI-2K scores. TPM, transcript per million. **p<0.01; ***p<0.001; ****p<0.0001
